# The genetic heterogeneity of Arab populations as inferred from *HLA* genes

**DOI:** 10.1371/journal.pone.0192269

**Published:** 2018-03-09

**Authors:** Abdelhafidh Hajjej, Wassim Y. Almawi, Antonio Arnaiz-Villena, Lasmar Hattab, Slama Hmida

**Affiliations:** 1 Department of Immunogenetics, National Blood Transfusion Center, Tunis, Tunisia; 2 Department of Medicine, Harvard Medical School, Boston, MA, United States of America; 3 Department of Immunology, University Complutense, School of Medicine, Madrid Regional Blood Center, Madrid, Spain; 4 Department of Medical Analysis, Hospital of Gabes (Ghannouch), Gabes, Tunisia; University of Michigan, UNITED STATES

## Abstract

This is the first genetic anthropology study on Arabs in MENA (Middle East and North Africa) region. The present meta-analysis included 100 populations from 36 Arab and non-Arab communities, comprising 16,006 individuals, and evaluates the genetic profile of Arabs using *HLA* class I (*A*, *B*) and class II (*DRB1*, *DQB1*) genes. A total of 56 Arab populations comprising 10,283 individuals were selected from several databases, and were compared with 44 Mediterranean, Asian, and sub-Saharan populations. The most frequent alleles in Arabs are A*01, A*02, B*35, B*51, DRB1*03:*01*, DRB1*07:*01*, DQB1*02:*01*, and DQB1*03:*01*, while DRB1*03:01-DQB1*02:*01* and DRB1*07:01-DQB1*02:*02* are the most frequent class II haplotypes. Dendrograms, correspondence analyses, genetic distances, and haplotype analysis indicate that Arabs could be stratified into four groups. The first consists of North Africans (Algerians, Tunisians, Moroccans, and Libyans), and the first Arabian Peninsula cluster (Saudis, Kuwaitis, and Yemenis), who appear to be related to Western Mediterraneans, including Iberians; this might be explained for a massive migration into these areas when Sahara underwent a relatively rapid desiccation, starting about 10,000 years BC. The second includes Levantine Arabs (Palestinians, Jordanians, Lebanese, and Syrians), along with Iraqi and Egyptians, who are related to Eastern Mediterraneans. The third comprises Sudanese and Comorians, who tend to cluster with Sub-Saharans. The fourth comprises the second Arabian Peninsula cluster, made up of Omanis, Emiratis, and Bahrainis. It is noteworthy that the two large minorities (Berbers and Kurds) are indigenous (autochthonous), and are not genetically different from “host” and neighboring populations. In conclusion, this study confirmed high genetic heterogeneity among present-day Arabs, and especially those of the Arabian Peninsula.

## Introduction

The human leukocyte antigens (*HLA*) system plays a key role in self-nonself recognition, and is divided into class I (*HLA-A*, *-B*, and *-C*) and class II (*HLA-DP*, *-DQ*, and -*DR*) loci, and comprises 220 genes in a 3.6 Mb region found on the short arm of chromosome 6. *HLA* system is highly polymorphic, and in excess of 17,000 alleles were detected. For example, there are 4,828 B, 3,968 A, and 3,579 C class I alleles, compared with 2,103 DRB1, and 1,142 *DQB1* class II alleles. Several *HLA* alleles were associated with various auto-immune and infectious diseases [1]. *HLA* class I and class II loci are characterized by high (80–90%) heterozygosity, and thus constitute reliable genetic markers for phylogenetic study, and thus are useful for anthropological studies.

Population studies confirmed varied frequencies of *HLA* alleles and haplotypes according to ethnicity and geographic origin. Given the codominant nature of the expression of *HLA* markers, this enables distinguishing between heterozygotes from homozygotes, hence allowing assignment of genotypes and allele frequencies [2]. Linkage disequilibrium (LD) analysis between *HLA* alleles identified the number of generations in-between two closely related populations from the time of their separation. Diversity in haplotype distribution, allele frequency, and LD analysis reflect the extent of variation between closely related populations. Allele frequency-based genetic distance analysis allows for construction of phylogenetic tree (Dendrograms), so as to infer relative estimate of the time that elapsed since the populations existed as single cohesive units [3–6].

Arabs are a major panethnic group, and their union, Arab League, is a cultural and ethnic union of 22 member states. As of 2013, nationals of the Arab League countries are 357 millions, who populate an area of 13 million km^2^, straddling Africa and Asia [7]. Ethnic, religious, and linguistic diversity (triple heterogeneity) characterize Arabs. Most Arabs follow Islam, and Christianity is the second largest religion, with over 15 million Christians. There are also smaller but significant religious minorities (as Druze, Jews), and a number of non-Arab ethnic minorities (as Berbers, Kurds) [7, 8].

The history of Arabs extends from circa 1200 BC when Southern Arabian Peninsula was ruled by three successive civilizations: Mineans, who established their capital Karna (1200–650 BC), Sabeans in Marib (1000 BC—570 AD), and the Himyarite (2nd-6th centuries AD) in Dhafar (Oman) [9–11]. These civilizations were built by authentic Yemeni tribes. The kingdom of Kinda was established in Central Arabia in 4th-early 6th century AD, while Dilmun civilization was founded in Eastern Arabia. In 3rd century AD, East African Kingdom of Aksum extended into Yemen and Western Saudi Arabia [12]. In addition, the Lakhmids (Yemeni origin), established a dynasty which ruled part of present-day Iraq and Syria in 300–602 AD [10, 13, 14]. The Arab Christian Ghassanids (220–638 AD), originating from Southern Arabia, migrated in 3rd century to Jordan, where they established their kingdom that extended from Syria to Yathrib (Saudi Arabia)[12.^13^]. Islam was introduced in 610 AD to Arabian Peninsula. Shortly thereafter, Arabian tribes were united as a single Islamic state in the Arabian Peninsula, which was spear-headed by the Islamic prophet Muhammad. This Islamic state progressively grew in area, and in types and numbers of populations, and extended from Andalusia (Spain) to the west, to Indus in the east [14].

Subsequent spread of Islam involved swift invasion of Persia (637-651AD), Iraq, Levant, and Egypt (639 AD), which extended into North Africa (640–709), and to Spain, Portugal, and France (Poitiers) in 8^th^ century AD. Eastwards, Arab expansion to Central Asia, Bukhara (Uzbekistan), Afghanistan (637–709), and the Indus border (664–712) followed. Northwards, Arab invaders were in contact with the Byzantine Empire, and the Caspian and Caucasus to the north [15, 16]. With the Islamic expansion from 7th century, social and political groups were gradually Arabized. The spreading of Arab-Muslim culture was at the expense of local languages (as Berber, Kurdish), especially in Middle East and North Africa, resulting in the Arabized population speaking variants of Arabic, mixed with original languages (*dialect*). The extent of gene Arab exchange with these autochthonous groups is undetermined but is thought to be lower than religious/cultural influence.

Given the large number of conquests, Arabs were in contact with different ethnicities residing on a vast area stretching from Mauritania (West Africa) to the western China border (East Asia). This suggests that cultural and perhaps genetic relationships were established with these ethnic groups. This work aims to study the *HLA* distribution in North African and Oriental Arab populations, and compare them to neighboring populations (Sub-Saharans Africans, Europeans, and Asians).

## Populations and methods

### Search strategy

Datasets of *HLA* allele frequencies were collected from a systematic review performed per Preferred Reporting Items for Systematic Reviews and Meta-analyses (PRISMA) criteria [Only the criteria from 1–10, 17, and 26 are applicable to this type of study (S1 Checklist)] [17]. PubMed, ScienceDirect, AlleleFrequencies.net, and ResearchGate databases were searched for all papers on *HLA* polymorphism, and *HLA* disease associations in Arabs. This systematic literature search covering published papers up to May 31, 2017 was conducted by two investigators (H.A and H.L); the search terms used were: ‘*HLA* Arabs’, or ‘Human Leukocyte Antigen Arabs’. A search per country followed: ‘*HLA* Tunisians’, ‘*HLA* Saudis’, and so on. This was repeated for remaining countries, which resulted in excess of 50 keywords used. A database from International Histocompatibility Workshops was also used. Some authors were also contacted by e-mail, or through ResearchGate, requesting information and missing data. While most datasets were taken from studies with an explicit anthropological focus, control groups from case-control disease studies were also used. There was no language restriction used for this search.

### Inclusion and exclusion criteria

All included studies met the following criteria. *HLA* allele frequencies must be obtained by molecular typing, and that subjects should be typed for at least one of the following: *HLA-A*, *HLA-B*, *HLA-DRB1*, and *HLA-DQB1*. Publications were excluded in case of serological data; sample size less than 35 individuals, typed individuals (or controls) were either related and not randomly selected, presentation of duplicate data sets. Studies were also excluded if they presented incomplete/partial allele frequencies, or there were significant ambiguities in the typing.

### Data extraction

Studies were independently selected by two authors (H.A and H.L). An external referee was invited in case of disagreements not resolved by both reviewers. Data extracted from selected papers included publication year, study type (anthropology, association), sample size, *HLA-A*, *-B*, *-C*, *-DRB1*, and *-DQB1* allele frequencies, haplotype frequencies, region, country, and typed loci.

### Statistical analysis

A three-dimensional correspondence analysis and bi-dimensional representation were performed using VISTA V5.02 software [18]. Phylogenetic trees were constructed based on allele frequencies using the Neighbor-Joining (NJ) method [19], and standard genetic distances (SGD) [20], using DISPAN software containing GNKDST and TREEVIEW software [21, 22].

## Results

### Study flow

**Fig 1:**
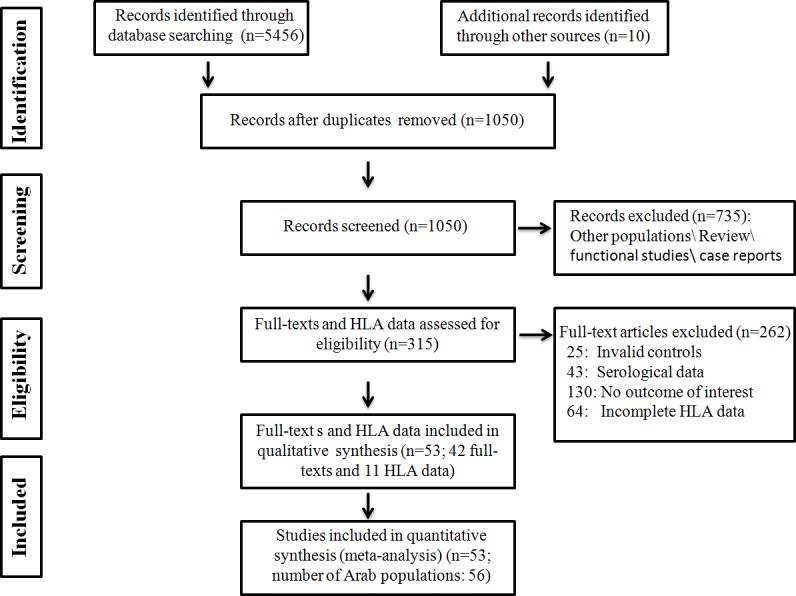
Flow diagram of the study selection process.

**Fig 2:**
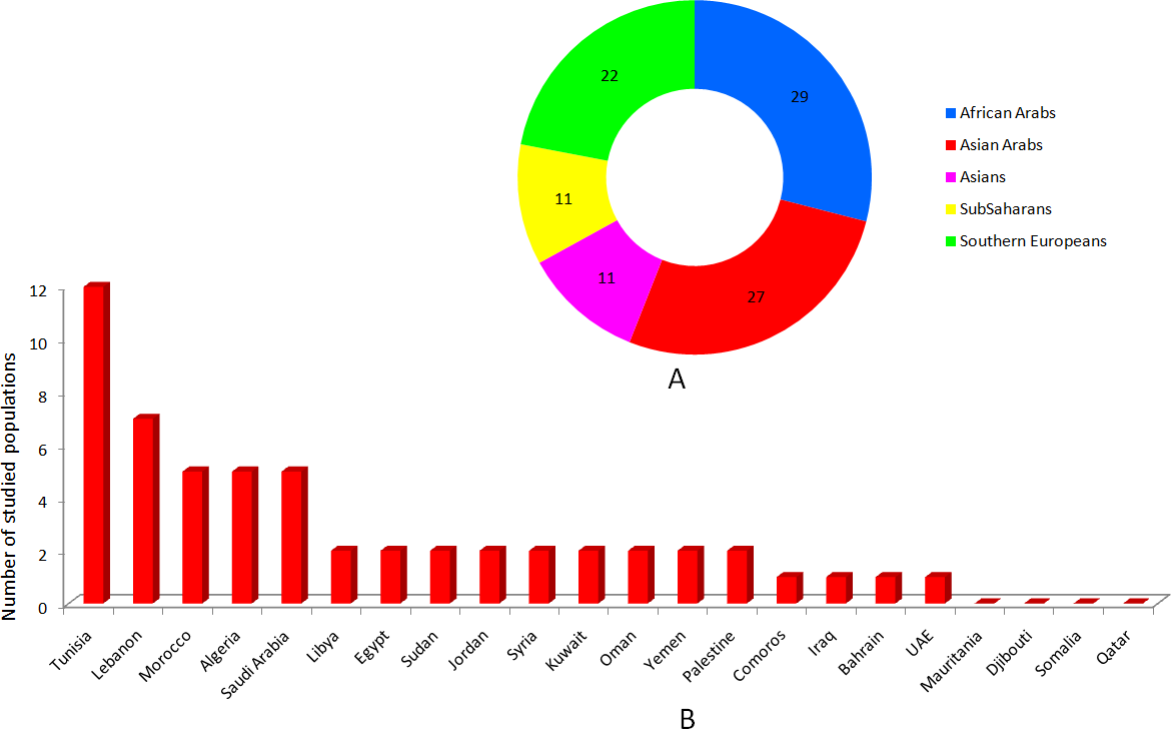
The distribution of studied populations by region (A) and country (B).

The use of more than fifty key words allowed identification of 5,456 papers and *HLA* datasets, of which 315 were deemed relevant to the study. Of these, 42 articles and 11 *HLA* datasets containing information on 56 Arab populations, and meeting the study criteria, were included. The study flow is illustrated in Fig 1. In addition, 20 articles and 18 *HLA* datasets which meet the criteria of this study, containing complete information on 44 other populations were selected, but without going through systematic review. The populations used in the comparison were chosen mainly from neighboring Arab countries. This study relied on a database consisting of 100 populations (of which data of 11 populations were extracted from association studies) from 36 countries Arab and worldwide countries, and belonging to Asia, Europe, and Africa. The distribution of populations by region is illustrated in Fig 2A. These populations represent allele frequency data for 16,006 individuals (160.06 individuals/population), and from 63 references.

### Selected populations

#### Arab populations

The 42 articles and 11 *HLA* datasets (http://www.allelefrequencies.net) selected provided information on 56 populations (Table 1), comprising 10,283 individuals [23–67]. The 56 different ethnic and religious populations were selected from 18 Arab countries. There were no reliable *HLA* data for the remaining countries (Somalia, Djibouti, Mauritania, and Qatar) (Fig 2B). The studied populations are divided into 29 African (26 North Africans and 3 Sub-Saharans), and 27 Asian populations (13 Levantines, and 14 Arabian Peninsula). With the exception of 8 populations [28, 38, 47, 48, 50, 52, 53, 55], where *HLA* data were extracted from association studies, the 50-remaining studies were extracted from anthropological ones.

**Table 1:** List of Arab populations used in the present work.

N^o^	Populations	Symbols	Size	References	N^o^	Populations	Symbols	Size	References
1	Algiers	Alg	102	[67]	29	Comorians	Com	117	[43]
2	Algerians-B	Alg-B	97	[23]	30	Jordanians	Jor	146	[31]
3	Algerians-A	Alg-A	132	[48]	31	Jordanians-A	Jor-A	1254	[46]
4	Algerians-Oran	Ora	100	[23]	32	Syrians	Syr	200	[47]
5	Gabesians	Gab	77	[59]	33	Syrians-A	Syr-A	225	[58]
6	Gabesians-A	Gab-A	96	[40]	34	Lebanese	Leb	95	[35]
7	Ghannouchians	Gha	82	[33]	35	Lebanese-A	Leb-A	1123	[45]
8	Berbers-Jerba	Ber-J	55	[40]	36	Lebanese-B	Leb-B	191	[44]
9	Berbers-Matmata	Ber-M	81	[40]	37	Lebanese-Armen	Leb-Ar	368	[27]
10	Berbers-Zrawa	Ber-Z	70	[24]	38	Lebanese-KZ	Leb-Kz	93	[39]
11	Tunisians	Tun	376	[61]	39	Lebanese-NS	Leb-Ns	59	[39]
12	Tunisians-A	Tun-A	80	[60]	40	Lebanese-Yohmor	Leb-Y	75	[39]
13	Tunisians-B	Tun-B	101	[34]	41	Palestinians	Pal	165	[29]
14	Tunisians-C	Tun-C	100	[63]	42	Palestinians-A	Pal-A	109	[36]
15	Tunisians-M	Tun-M	123	[26]	43	Saudis	Sau	105	[28]
16	Southern Tunisians	Tun-S	250	[62]	44	Saudis-A	Sau-A	213	[23]
17	Libyans	Lib	118	[32]	45	Saudis-B	Sau-B	158	[49]
18	Libyans-Jews	Lib-J	119	[36]	46	Saudis-C	Sau-C	499	[23]
19	Berbers-Metelsa	Ber-Me	99	[64]	47	Saudis-D	Sau-D	383	[50]
20	Moroccans	Mor	96	[25]	48	Omanis-A	Oma-A	259	[30] [51]
21	Moroccans-A	Mor-A	110	[42]	49	Kuwaitis	Kuw	212	[52]
22	Moroccans-Agadir	Mor-Ag	98	[37]	50	Kuwaitis-A	Kuw-A	114	[53]
23	Moroccans-Chaouya	Mor-Ch	98	[65]	51	Bahrainis	Bah	72	[35]
24	Moroccans-Jews	Mor-J	94	[66]	52	Emiratis	Emi	373	[23]
25	Egyptians	Egy	101	[39]	53	Iraq kurds	Ira-K	209	[54]
26	Egyptians-A	Egy-A	121	[38]	54	Yemenite-Jews	Yem-J	76	[36]
27	Sudanese	Sud	200	[23]	55	Yemen-sana'a	Yem	50	[55]
28	Sudanese-Nuba	Sud-N	46	[23]	56	Omanis	Oma	118	[56] [57]

#### Neighboring populations

Forty-four worldwide populations [23, 34, 39, 66, 68–85] comprising 5,723 individuals, were selected from 18 countries in three continents, using the same criteria previously described (Table 2). These comprised 22 European, 11 non-Arab Asian, and 11 Sub-Saharan African populations. Of the 11 Asian populations, there were two Arab minorities living in Iran (Khuzestan and Famoori).

Data of only three populations [74, 75, 84] were extracted from association studies. These populations were typed for at least *HLA-A*, *-B*, *-DRB1*, or *DQB1*.

**Table 2:** Worldwide populations included in the meta-analysis.

N^o^	Populations	Symbols	Size	References	N^o^	Populations	Symbols	Size	References
1	Spaniards	Spa	176	[41]	23	Mossi	Mos	42	[39]
2	Portuguese	Por	118	[39]	24	Mandenka	Mad	200	[39]
3	Murcians	Mur	173	[80]	25	Amhara	Amh	98	[39]
4	Italians	Ita	284	[68]	26	Bubi	Bub	101	[39]
5	Basques-A	Bas-A	82	[41]	27	Congolese	Con	85	[72]
6	Basques-Arratia	Bas-Ar	83	[77]	28	Fulani	Ful	38	[39]
7	Basques-B	Bas-B	99	[70]	29	Gabonese	Gab	167	[85]
8	French	Fre	179	[68]	30	Nigerians	Nig	258	[23]
9	French-Rennes	Fre-R	200	[34]	31	Oromo	Oro	83	[39]
10	Balearic	Bal	90	[71]	32	Rimaibe	Rim	39	[39]
11	Corsica	Cor	100	[71]	33	Senegalese	Sen	177	[39]
12	Sardinians	Sar	91	[68]	34	Famoori Arabs	Fam	84	[73]
13	Ashkenazi-Jews	Ash-J	132	[66]	35	India-Northeast	Ind-N	188	[83]
14	Greeks-A	Gre-A	96	[39]	36	Indians-Delhi	Ind-D	112	[84]
15	Greeks-B	Gre-B	101	[39]	37	Iranian-Jews	Ira-J	91	[73]
16	Greeks-C	Gre-C	98	[39]	38	Iranians	Ira	120	[74]
17	Greeks-D	Gre-D	242	[23]	39	Iranians-A	Ira-A	100	[75]
18	Macedonians	Mac	172	[78]	40	Iranians-Azeri	Ira-Az	100	[81]
19	Turks	Tur	250	[23]	41	Iranians-Kurd	Ira-k	100	[81]
20	Turks-A	Tur-A	228	[79]	42	Khuzestani Arabs	Khu	50	[73]
21	Albanians	Alb	160	[76]	43	Pakistanis-Pathan	Pak-P	100	[82]
22	Cretans	Cre	135	[69]	44	Pakistanis-Sindh	Pak-S	101	[82]

### HLA allele frequencies features of Arab populations

Table 3 shows the most frequent *HLA-A* and *-B* alleles in Arab populations. A*02 was the most prevalent allele, and its frequency exceeded 25% in some populations, such as Saudis (30.4%) [23], Tunisian Berbers of Zrawa (29.3%) [24], Moroccans (26.2%) [25], and Sudanese (25.9%) [23]. A*01, *03, *24, *30, and *68 alleles were also common in most Arab populations. For example, the highest frequency of A*01 was seen in Tunisians (15%) [26] and Moroccans (14.8%) [25], while A*03 was prevalent among Iraqi Kurds (15.1%) [23], and A*30 was prevalent among Sudanese (17.6%) [23]. In addition, A*24 was common among Lebanese-Armenians (17.3%) [27], while A*68 was prevalent in Saudis (10.5%) [28]. In contrast, A*25, *28, *34, *36, *43, *66, *69, *74, and *80 are rare among Arabs. It is noteworthy that A*34, described as rare allele among Arabs, is found at a high frequency (22.2%) in Tunisian Berbers from Zrawa [24], the highest reported for any population worldwide.

**Table 3:** Most frequent HLA-A* and–B* alleles in Arab populations.

***HLA-A***	A*01		A*02		A*03		A*24		A*30		A*68	
	Population	%	Population	%	Population	%	Population	%	Population	%	Population	%
	Tun-M	15.0	Sau-D	30.4	Ira-k	15.1	Leb-Ar	17.3	Sud	17.6	Sau	10.5
	Mor	14.8	Ber-Z	29.3	Leb-Ar	14.0	Gha	15.2	Mor-C	13.0	Tun-M	09.4
	Jor-A	14.7	Mor	26.2	Pal	10.7	Ira-k	13.9	Tun-A	11.8	Mor	09.3
	Ira-k	13.2	sud	25.9	Lib	10.3	Sau-B	13.3	Jor	11.5	Alg-K	08.6
	Pal	12.5	Emi	25.2	Mor-A	10.0	Jor-A	10.7	Alg-K	10.2	sud	08.5
	Leb-A	12.2	Oma	24.9	Alg-K	09.3	Pal	10.1	Sau-B	10.2	Emi	08.4
	Sau-A	12.2	Alg	24.6	Jor-A	09.1	Alg	09.4	Pal	08.4	Lib	08.2
	Alg	11.9	Lib	23.5	Emi	09.1	Lib	09.3	Oma-A	07.5	Jor	07.6
	Lib	11.5	Jor-A	22.0	Sau-A	08.9	Mor	07.3	Leb-A	06.7	Oma-A	07.1
	Oma	07.2	pal	20.5	Gab	07.7	Oma	06.3	Lib	06.4	Leb-A	05.1
	Sud	06.5	Leb-A	18.7	Sud	07.1	Sud	06.1	Emi	05.0	Ira-k	03.8
	Emi	06.2	Ira-k	17.0	Oma	06.4	Emi	05.2	Ira-k	03.8	Pal	03.6
***HLA-B***	B*07		B*08		B*35		B*44		B*50		B*51	
	Population	%	Population	%	Population	%	Population	%	Population	%	Population	%
	Jor	27.1	Oma	11.0	Pal	20.3	Ber-Z	32.8	Sau-D	18.8	Sau-C	19.3
	Sau-A	11.7	sau-B	10.1	Leb-Ar	19.8	Ira-k	10.3	Lib	16.1	Oma	17.5
	Mor	09.0	Emi	08.6	Ira-k	15.6	Mor-C	10.2	Ber-Z	15.7	Emi	156
	Lib	07.7	Gha	08.5	Oma-A	15.3	pal	09.6	Tun-S	14.2	Ira-K	15.6
	Tun-A	07.5	Ira-k	07.2	Jor-A	14.9	Alg	08.8	Mor-C	12.5	Gha	12.2
	Alg-k	07.1	Lib	06.4	Emir	11.1	Leb-Ar	08.4	Emi	09.4	Leb-Ar	12.1
	Leb-Ar	04.5	Mor-C	06.2	Alg	10.3	Lib	07.6	Jor-A	06.4	Lib	11.1
	Ira-k	04.1	Jor	04.7	Lib	10.1	Jor-A	05.6	Pal	05.8	Jor-A	10.3
	Oma-A	03.1	Sud	04.0	Tun-M	09.8	Sau-D	03.5	Leb-Ar	05.2	Sud	07.8
	Sud	02.8	Alg	03.5	Sau	08.6	Sud	02.3	Alg	05.1	Mor	07.4
	Emi	02.4	Leb-Ar	03.0	Mor-C	06.9	Emi	02.3	Oma-A	04.2	Pal	06.4
	Pal	01.8	Pal	02.7	sud	06.1	Oma-A	02.1	Sud	02.5	Alg-k	04.7

Only one population per country is illustrated; the frequencies are ranked from highest to lowest for each allele; to identify the population and country see Table 1

Results of *HLA-B* locus are presented in Table 3. B*35 was the most frequent B* allele in Palestinians (20.3%) [29] and Lebanese-Armenians (19.8%) [27]. B*35 was found at varied frequencies in Iraqi Kurds (15.6%) [23], Omanis (15.3%) [30], Jordanians (14.9%) [31], and Arab Emirati (11.1%) [23] populations. B*51 was the second most frequent allele, and high frequencies were recorded for Saudis (19.3%) [23], Omanis (17.5%) [30], and Arab Emirati (15.6%) [23] populations. B*50 was also a frequent B* allele in most Arabs, including Saudis (18.8%) [23], and Libyans (16.1%) [31], along with B*08, and B*44 among the Tunisian Berbers of Zrawa (32.8%) [24], the latter being the highest frequency worldwide. Similarly, the frequency of B*27 is the highest among Jordanians (27.1%) [31]. In contrast, B*37, *42, *46, *47, *48, *54, *59, *67, and *78 alleles are extremely rare or virtually in all Arab populations.

The most common *DRB1* and *DQB1* alleles among Arabs are shown in Table 4. DRB1*07:01 was the most frequent allele among Tunisians from Ghannouch (28.6%) [33], Jordanians (26.9%) [31], and Saudis (26.6%) [23], while Egyptians (8.3%) and Sudanese had the lowest frequencies of DRB1*07:*01*. DRB1*03:*01* was the second most frequent DRB1* allele in some Arabs, such as Tunisians of Tunis (21.9%) [34] and Moroccans of Metelsa (20.2%) [23], but rare in Jordanians (2.4%) [31]. DRB1*11:*01* was also frequent among some Arabs, such as Lebanese (36.8%) [35], but rare among Saudis (4.8%) and Moroccans of Chayoua (2.5%) [23]. Furthermore, DRB1*13:*01*, *13:*02*, and *15:*01* alleles are relatively frequent among Arabs. High frequency of DRB1*13:*01* were recorded for Sudanese (23.3%), while DRB1*13:*02* was virtually absent in Bahraini [35] and Sudanese [23]. All DRB1*09, *12, and *14 subtypes are extremely rare among Arabs. In addition, DRB1*16 subtypes are rare in all Arab populations except for Bahrain, where DRB1*16:*01* is found at a high frequency (13.9%) [35].Haut du formulaire.

**Table 4:** Most frequent HLA-DRB1* and–DQB1* alleles in Arab populations.

HLA-DRB1*	***03*:*01***		***07*:*01***		***11*:*01***		***13*:*01***		***13*:*02***		***15*:*01***	
	Population	%	Population	%	Population	%	Population	%	Population	%	Population	%
	Tun-B	21.9	Gha	28.6	Leb	36.8	Sud	23.3	Mor-Me	11.1	Alg-B	13.4
	Mor-Me	20.2	Jor	26.9	Bah	16.0	Sau-A	10.6	Lib	09.3	Mor-c	12.6
	Sau-B	16.5	Sau-B	26.6	Egy-A	13.2	Ber-M	08.0	Sau-A	08.9	Ber-Z	11.4
	Ora	15.1	Yem-J	22.1	Gab-A	11.2	Leb-B	06.8	Egy-A	07.4	Jor	09.0
	Bah	13.9	Mor-Ag	20.5	Pal	10.0	Alg-B	05.6	Tun-C	06.7	Sau-A	08.9
	Sud	13.8	Lib-Y	19.6	Ora	08.6	Lib	05.5	Leb-N	05.0	Bah	07.6
	Lib	13.6	Lib	17.0	Sud	08.3	Yem-J	05.4	Ora	04.5	Leb	04.7
	Yem-J	12.0	Alg-B	15.9	Jor	08.3	Egy-A	04.6	Yem-J	04.0	Lib	04.2
	Leb-B	09.6	Pal	12.7	Lib	05.1	Mor-Me	03.5	Pal	03.9	Pal	03.6
	Pal	07.6	Bah	09.0	Sau-A	04.8	Jor	02.1	Jor	00.3	Sud	03.3
	Egy-A	07.0	Egy-A	08.3	Yem-J	03.4	Bah	02.1	Sud	00.0	Egy-A	02.5
	Jor	02.4	Sud	07.8	Mor-C	02.5	Pal	00.9	Bah	00.0	Yem-J	02.0
HLA-DQB1*	***02*:*0X***		***03*:*01***		***03*:*02***		***05*:*01***		***06*:*02***		***06*:*03***	
	Population	%	Population	%	Population	%	Population	%	Population	%	Population	%
	Gha	40.1	Leb-NS	45.0	Gha	20.7	Bah	29.2	Mor-C	12.9	Egy-A	10.2
	Yem-J	39.1	Ora	35.1	Jor	17.8	Ber-J	22.7	Alg	12.8	Jor	08.3
	Mor-Ag	37.8	Lib-J	29.6	Pal	17.6	Leb	20.5	Egy-A	12.7	Ber-J	07.8
	Sau-B	37.3	Ber-J	27.4	Leb	16.8	Alg	13.9	Tun-A	12.6	Lib-J	07.4
	Jor	35.9	Pal	26.7	Yem-J	14.2	Mor-C	12.3	Jor	10.7	Yem-J	06.1
	Lib-J	33.3	Yem-J	19.1	Lib-J	13.0	Pal	11.8	Sau-B	05.1	Ora	04.3
	Bah	25.7	Bah	16.0	Alg	12.3	Sau-B	10.1	Pal	04.2	Sau-B	04.1
	ora	24.5	Mor-C	15.4	Mor-C	12.3	Jor	09.3	Leb-Y	03.7	Leb-Y	03.3
	Pal	20.9	Egy	11.9	Bah	09.7	Egy-A	08.5	Yem-J	02.0	Mor-C	01.8
	Leb-Y	20.0	Jor	10.0	Sau-B	08.9	Yem-J	06.1	Lib-J	00.8	Pal	01.2

Only one population per country is illustrated; the frequencies are ranked from highest to lowest for each allele; to identify the population and country see Table 1

DQB1*02:*0X* and *03:*01* alleles are the most frequent DQB1* in Arabs. The highest frequencies of DQB1*02:*0X* were reported for Tunisians (Ghannouch; 40.01%) [33], Yemenites-Jews (39.1%) [36], Moroccans (Agadir-Souss; 37.8%) [37] and Saudis (37.3%) [23], while the lowest frequency was found in Egyptians (6%) [38]. On the other hand, DQB1*03:*01* is very common among Lebanese (45%) [39] and Algerians (Oran; 35.1%) [23], but not Saudis (7.6%) [23]. DQB1*03:*02* and *05:*01* are also frequent in most Arabs, such as Tunisians (Ghannouch; 20.7%) [33], Jordanians (17.8%) [31], Palestinians (17.6%) [29] and Lebanese (16.8%) [35]. DQB1*05:*01* is frequent among Bahrainis (29.2%) [35], Tunisians (Berbers of Jerba; 22.7%) [40], and Lebanese (20.5%) [35]. Among DQB1*06 subtypes, DQB1*06:*02* and *06:*03* were the most frequent in most Arab populations, but absent in Bahrainis where DQB1*06:*01* is very frequent (13.20%) [35]. Furthermore, all DQB1*04 subtypes are rare among Arabs, particularly DQB1*04:*01* which is virtually absent, except in Egyptians (10.17%) [38]. The most common DQB1*04 subtype in Arabs is DQB1*04:*02*.

### Allelic comparison between Tunisians and other populations

Allelic comparisons were done at Neighbor-Joining, correspondence analysis, and standard genetic distances. Analyses were performed with Class I and Class II markers, and at generic and high-resolution levels to make the most of available data, and seeing that some of the populations included in these comparisons lack high-resolution data.

#### Neighbor-joining dendrograms

**Fig 3:**
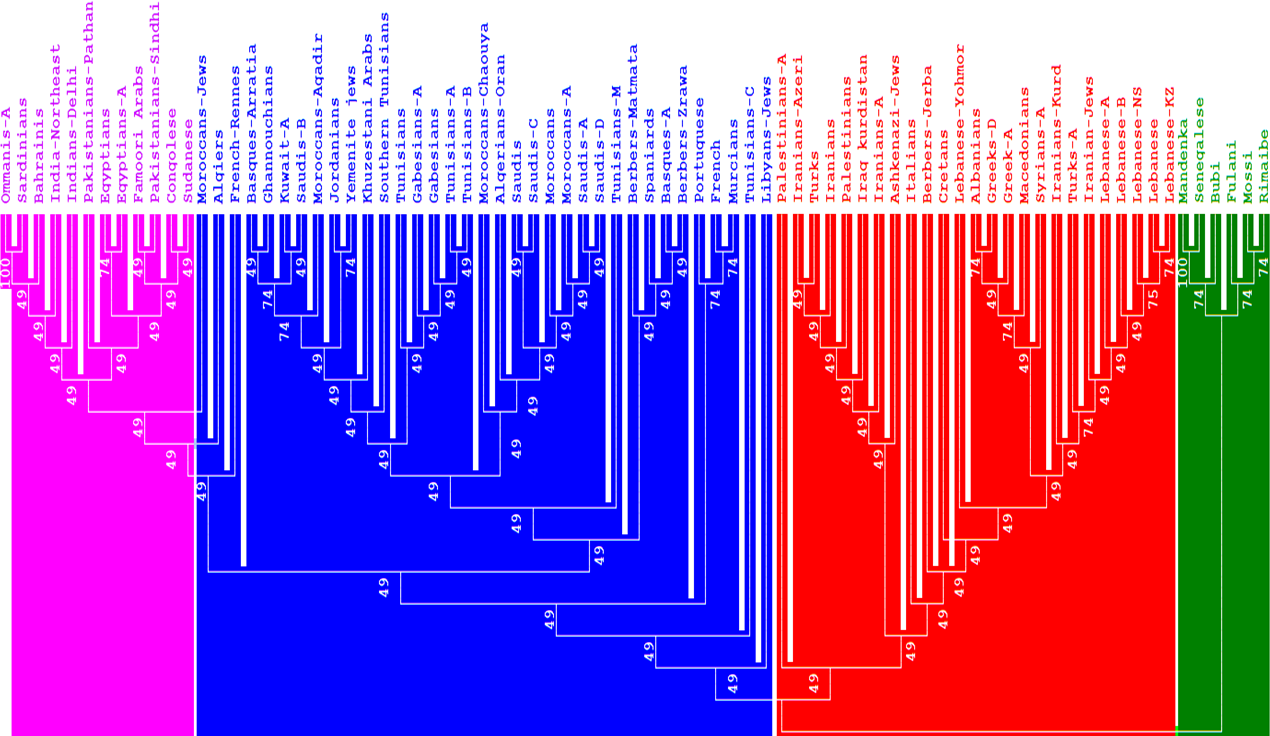
Neighbor-Joining dendrograms, based on Standard genetic distances (SGD), showing relatedness between Arabs and other populations using generic HLA-DRB1* and -DQB1* allele frequencies data. Populations’ data were taken from references detailed in Tables 1 and 2. Bootstrap values from 1.000 replicates are shown.

Comparison at the generic level was made using genetic distances based on DRB1* and DQB1* allelic frequencies. Four groups can be interpreted from Fig 3. The first group comprises North African Arabs (Tunisians, Algerians, Moroccans, Libyans), Western Mediterranean Europeans (Iberians, French), Arabian Peninsula Arabs (Saudis, Kuwaitis, Yemenis), and Arab minority of Iran (Khuzestani). The second group is formed by Eastern Mediterranean Europeans (Greeks, Cretans, Albanians, Turks, Macedonians), Italians, Levant Arabs (Palestinians, Lebanese, Syrians), Iraqi-Kurds, Tunisian Berbers (Djerba), and Iranians. The third group comprises Sub-Saharan Africans (Fulani, Mossi, Rimaibe, Bubi, Mandenka, and Senegalese). Omanis, Bahrainis, Egyptians, and Sudanese form a heterogeneous group containing Asians and Sub-Saharan Africans. Similar results but with notable differences, were observed in dendrograms built with standards genetic distances (SGD) based on generic *DRB1*(S1 Fig) and generic *B* loci (S2 Fig).

#### Correspondence analysis

**Fig 4:**
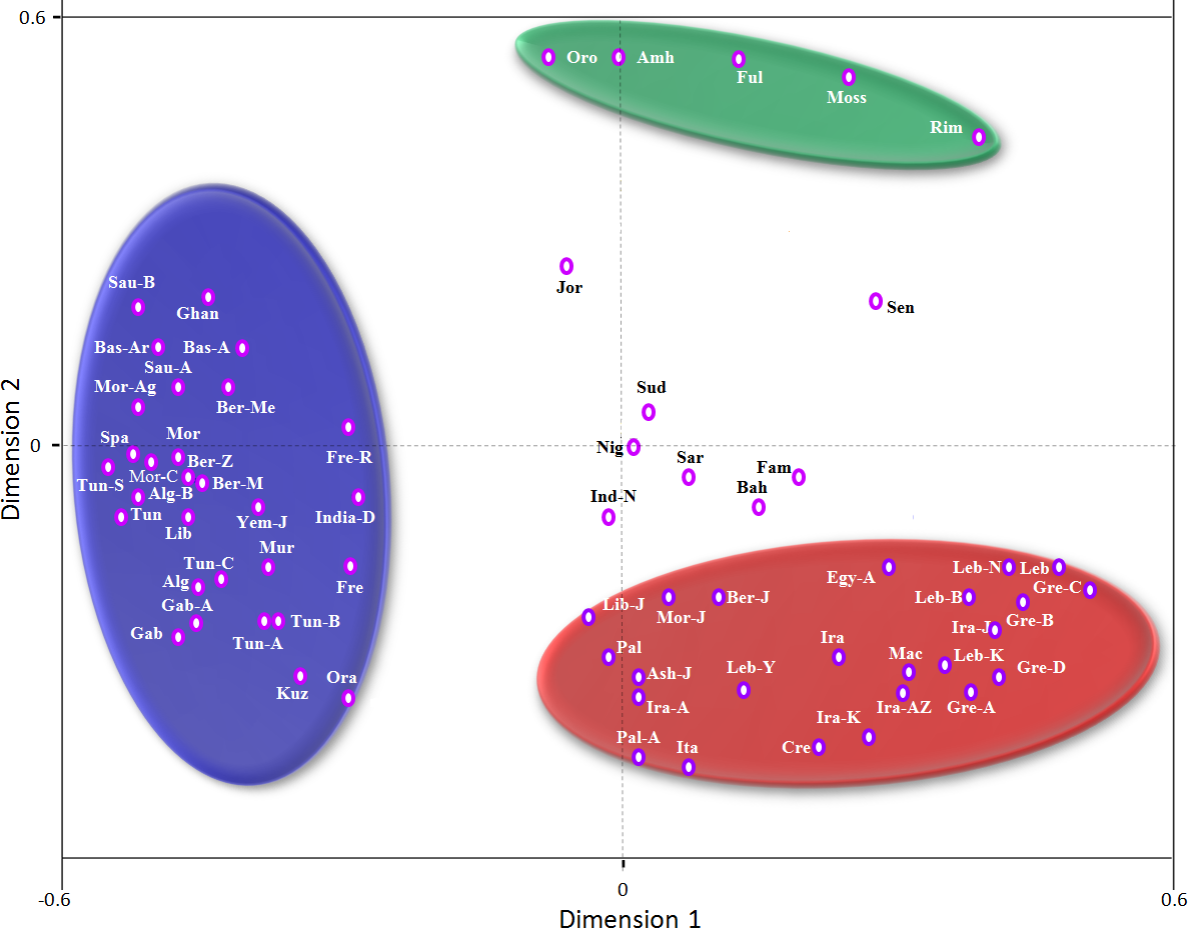
Correspondence analysis (bi-dimensional representation), based on the standard genetic distances, showing the relationship between Arabs and other populations according to high resolution HLA-DRB1* allele frequencies data. Only individuals with defined DRB1* subtypes are considered. Populations data were taken from references detailed in Tables 1 and 2.

**Fig 5:**
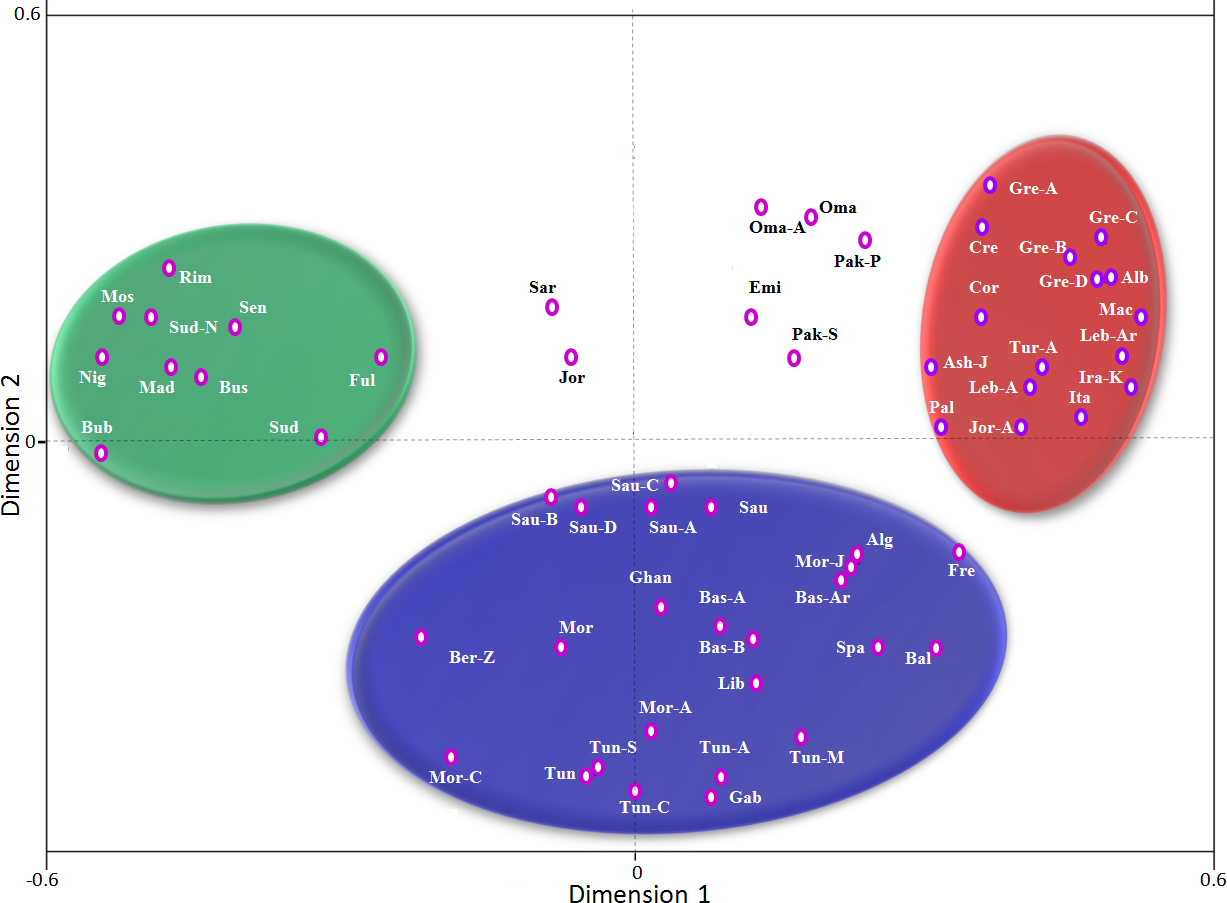
Correspondence analysis (bi-dimensional representation), based on the standard genetic distances, showing a global view of the relationship among Arabs and other populations according to generic HLA*-A and–B* allele frequencies data. Populations data were taken from references detailed in Tables 1 and 2.

High-resolution *DRB1* correspondence analysis (Fig 4) demonstrated the clustering of the studied populations into three groups. The first containing North Africans (Tunisians, Algerians, Moroccans, and Libyans), Iberians (Basques, Spaniards, Portuguese, Murcians), French, Saudis, Yeminis-Jews, and Khuzestani Arabs. The second contains Eastern Mediterraneans (Greeks, Cretans, Lebanese, Palestinians, and Macedonians), Berbers of Djerba, Italians, Iraqi-Kurds, Iranians, Egyptians, Ashkenazi-Jews, and Moroccan-Jews. The last cluster consists of Sub-Saharan populations. It should be noted that Jordanians, Bahrainis, and Sudanese were outside these main groups. Similarly, correspondence analysis using class I (*A* and *B*) identified three main clusters (Fig 5). The first cluster contained all Sub-Saharan Africans along with Sudanese. The second cluster contains Eastern Mediterranean populations (Albanians, Greeks, Cretans, Lebanese, Palestinians, and Macedonians), Italians, Iraqi-Kurds, Ashkenazi-Jews, and Jordanians-A. The last cluster includes North Africans (Tunisians, Algerians, Moroccans, and Libyans), Iberians (Basques, Spaniards), French, and Saudis.

**Fig 6:**
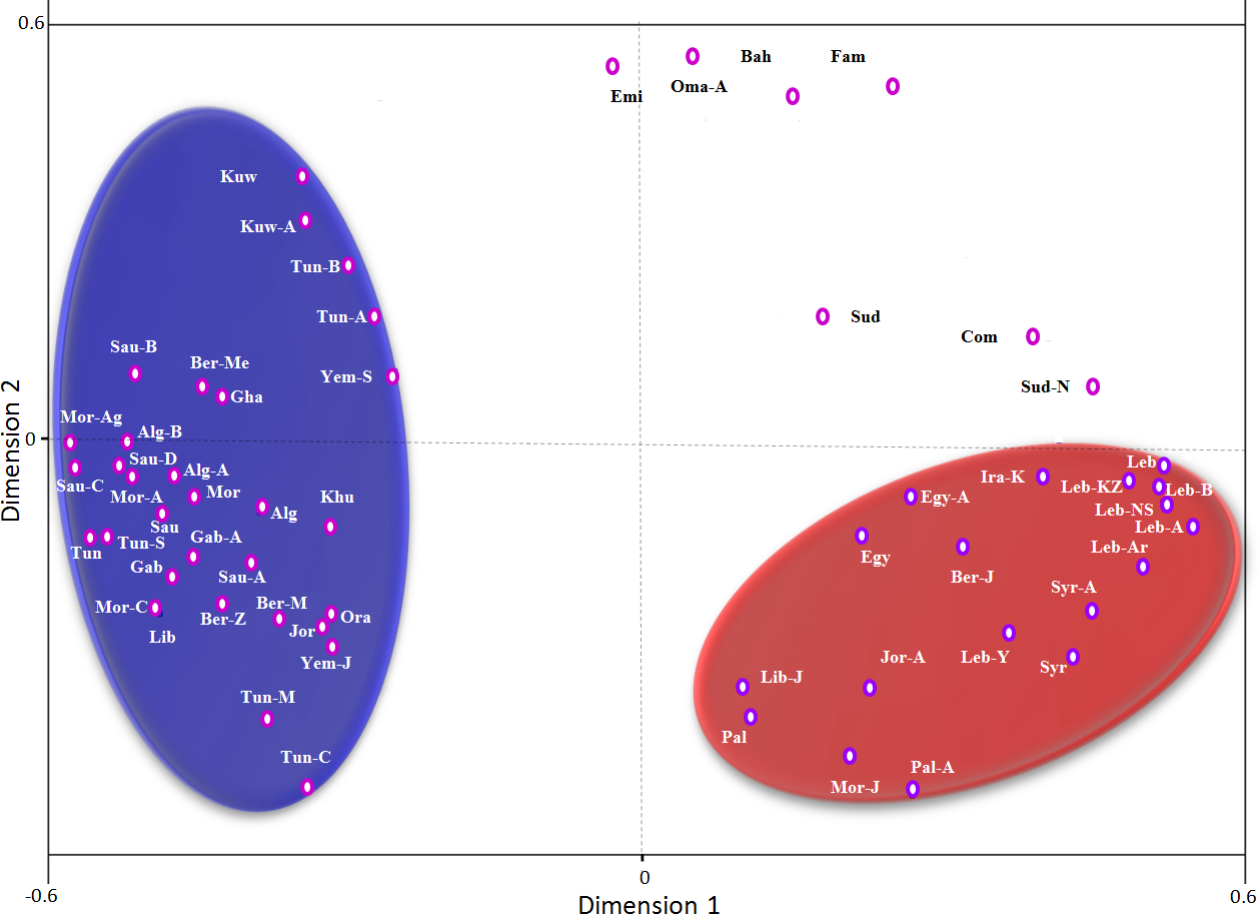
Correspondence analysis (bi-dimensional representation), based on the standard genetic distances, showing the relationship between different Arab populations according to generic HLA-DRB1* allele frequencies data. Populations data were taken from references detailed in Tables 1 and 2.

Correspondence analysis based on generic *DRB1* data, and using only Arab populations shows that Arabs can cluster into four groups (Fig 6). The first contains the North Africans (Tunisians, Algerians, Moroccans, and Libyans), Saudis, Yemenis, Kuwaitis, and Khuzestanis (Iranian Arabs). The second cluster includes the Arabs of Levant (Palestinians, Jordanians, Lebanese, Syrians), Egyptians, Iraqi Kurds, and Moroccans Jews. The third group consists of Bahrainis, Omanis, Emiratis and Famoori (Iranian Arab). The fourth is composed of Sudanese, Sudanese from Nuba, and Comorians.

#### Genetic distances

Table 5 illustrates standard genetic distances (SGD) between Arabs and other populations, using generic DRB1* allele frequencies. North Africans and Iberians are the closest to Saudis. Moroccans (Agadir, 0.0024), Basques-Ar (0.0057), and Tunisians-S.

**Table 5:** The closest populations to Arabs using standard genetic distances (SGD) based on HLA-DRB1* alleles.

**Saudis-B**		**Emiratis**		**Omanis-A**		**Sudanese**	
**Population**	**SGD**	**Population**	**SGD**	**Population**	**SGD**	**Population**	**SGD**
Moroccans-Ag	0.0024	Omanis-A	0.0411	Emirates	0.0411	Nigerians	0.0497
Basques-Ar	0.0057	Bahrain	0.0429	Sardinians	0.0939	Egyptians-A	0.0556
Tunisians-S	0.0124	Sardinians	0.0593	Bahrain	0.1327	Congolese	0.0594
Saudis-C	0.0160	Kuwaitis	0.0688	Kuwait	0.2014	Egyptians	0.0620
Ghanouchians	0.0203	Tunisians-B	0.1169	Famoori Arabs	0.2377	Mandenka	0.0908
Saudis	0.0258	Khuzestanis	0.1213	Macedonians	0.2461	Moroccans	0.0984
Tunisians	0.0272	Tunisians-A	0.1276	Tunisians-B	0.3071	Senegalese	0.1044
Kuwaitis-A	0.0312	Algerians-Oran	0.1371	Khuzestanis	0.3192	Bubi	0.1078
Khuzestanis	0.0349	Algerians-A	0.1407	Greeks-B	0.3197	Palestinians-A	0.1111
Spaniards	0.0354	Algerians-B	0.1612	Tunisians-A	0.3261	Pakistanis-S	0.1122
Saudis-D	0.0374	Algiers	0.1639	Kuwaitis-A	0.3544	Tunisians-A	0.1133
Gabesians	0.0377	Saudis-C	0.1746	Algerians-Oran	0.3600	Libyans	0.1197
Gabesians-A	0.0394	Macedonians	0.1756	Algerians-A	0.3639	Sudanese-Nuba	0.1234
Jordanians	0.0428	Gabesians	0.1820	Greeks-D	0.3657	Algerians-B	0.1315
Algerians-B	0.0433	Saudis-D	0.1820	Algerians-B	0.3867	Berbers-Matmata	0.1317
Basques-B	0.0449	Moroccans-Agadir	0.1830	Greeks-C	0.3927	Algerians-A	0.1407
Saudis-A	0.0450	Kuwaitis-A	0.1837	Turks	0.3944	Berbers-Zrawa	0.1409
Algerians-A	0.0497	Famoori Arabs	0.1894	Saudis-C	0.3984	Gabesians	0.1413
Tunisians-C	0.0533	Moroccans-A	0.1900	Algiers	0.4027	Jordanians-A	0.1434
Yemenite-J	0.0536	Gabesians-A	0.1908	Albanians	0.4034	Gabesians-A	0.1442
**Khuzestanis**		**Tunisians**		**Syrians-A**		**Comorians**	
**Population**	**SGD**	**Population**	**SGD**	**Population**	**SGD**	**Population**	**SGD**
Gabesians	-0.0086	Gabesians	-0.0139	Cretans	-0.0001	Congolese	0.0519
Orans	-0.0074	Gabesians-A	-0.0081	Lebanese-Ar	0.0050	Nigerians	0.0828
Gabesians-A	-0.0025	Moroccans-Agadir	-0.0080	Syrians	0.0076	Greeks-A	0.0836
Algerians-A	-0.0015	Southern Tunisians	-0.0062	Iranians-Kurd	0.0100	Gabonese	0.0904
Moroccans-Ag	0.0106	Algerians-A	-0.0055	Lebanese-A	0.0149	Iranians-A	0.0947
Tunisians-S	0.0140	Moroccans-A	0.0010	Lebanese-Y	0.0151	Egyptians-A	0.1090
Tunisians	0.0161	Algerians-B	0.0019	Iranians	0.0159	Iranians	0.1184
Tunisians-C	0.0195	Berbers-Zrawa	0.0027	Lebanese-B	0.0161	Italians	0.1222
Yemenite-J	0.0217	Libyans	0.0028	Iranians-Azeri	0.0185	Iranians-Azeri	0.1394
Tunisians-M	0.0225	Algerians-Oran	0.0033	Turks	0.0192	Iranians-Kurd	0.1418
Saudis-C	0.0231	Tunisians-M	0.0038	Iraq kurdistan	0.0198	Albanians	0.1426
Spaniards	0.0291	Saudis-C	0.0061	Ashkenazi-Jews	0.0222	Turks	0.1428
Saudis	0.0324	Tunisians-C	0.0083	Iranians-A	0.0223	Syrians	0.1470
Saudis-B	0.0349	Algiers	0.0103	Palestinians-A	0.0228	Cretans	0.1483
Algerians-B	0.0353	Berbers-Matmata	0.0106	Italians	0.0241	Egyptians	0.1483
Tunisians-B	0.0422	Moroccans-Chaouya	0.0111	Turks-A	0.0288	Greeks-C	0.1487
Indians-Delhi	0.0454	Spaniards	0.0126	Lebanese	0.0320	Palestinians-A	0.1559
Algiers	0.0461	Moroccans	0.0144	Jordanians-A	0.0355	Iraq Kurdistan	0.1564
Basques-Ar	0.0471	Saudis-D	0.0159	Lebanese-KZ	0.0368	Greeks-D	0.1594
Libyans	0.0485	Khuzestani Arabs	0.0161	Greeks-A	0.0407	Syrians-A	0.1617

(0.0124) had the closest genetic distances from Saudis, while Emiratis were closely related to Omanis (0.0411), Bahrainis (0.0429), Sardinians (0.0593), and Kuwaitis (0.0688). On the other hand, Sudanese are related to Sub-Saharans, including Nigerians (0.0497), Congolese (0.0594), and Egyptians (0.556).

Syrians are genetically close to Eastern Mediterranean, as Cretans (-0.0001) and Lebanese Armenians (0.0050), while Tunisians are closed to Western Mediterraneans as North Africans and Iberians, and Saudis. The populations most related to Tunisians are the other Tunisian populations (Gabesians, -0.0139), Moroccans (Agadir; -0.0080), and Algerians (-0.0055). Sub-Saharans such as Congolese (0.0519) and Nigerians (0.0828), and Greeks (0.0836) showed the closest genetic distances to Comorians. It is noteworthy that Arab minority in Khuzestan (Iran) displayed close relatedness with North Africans [as Gabesians from Tunisia (-0.0086) and Orans from Algeria], and Saudis (0.0231).

### *HLA* Class I and Class II haplotype

#### *HLA-A-B* haplotypes

*HLA A-B* haplotypic data are extremely rare in Arabs. The most frequent *A-B* haplotypes in Arabs are shown in Table 6. A*02:01-B*50:*01* (9.0%) and A*02:01-B*44:*02/03* (7.5%) were the haplotypes with the highest frequencies in Berbers of Zrawa. Diversity in *A-B* haplotype frequencies are found among Arabs, hence demonstrating comparable frequencies of *A-B* haplotype in Arab populations, which did not exceed 5.3% in Gabesians (Tunisia). For example, while A*34:02-B*08:*01* and A*29:01-B*45:*01* characterize Tunisians, A*01-B*57(02.*9%)*, A*30-B*18 (01.50%), and A*33:01-B*14:*01* (02.50%) characterize Algerians. Several haplotypes identified in Arabs were also seen in other Mediterraneans. For example, A*32:01-B*40:*02* was seen in Greeks (2%) [39] and Spaniards (0.5%) [41], while A*02:01-B*50:*01* was seen in Italians (2%) [68], Portuguese (3%) [39], and Moroccan Jews (3%) [66]. A*24:02-B*08:*01* (4.75%) and A*30:02-B*53:*01* (3.48%) were only identified in Saudis.

**Table 6:** Most frequent (%) *HLA* Class I (*A-B*) two-locus haplotypes with significant linkage disequilibrium (P<0.05) in Arabs.

A-B haplotype	Tun	Saudi-B	Alg	Mor-Ch	Mor-a	Ber-Z	Lib	Gab
*01*:*01–50*:*01*	-	-	-	04.10	-	-	-	-
*01–57*	-	-	02.90	-	-	-	-	-
*02*:*01–07*:*02*	-	-	-	-	-	-	02.97	-
*02*:*01–44*:*02/03*	03.86	-	-	02.10^a^	02.95^c^	07.50^b^	-	05.26
*02*:*01–50*:*01*	03.30	-	-	-	01.99^d^	09.01	-	-
*02*:*01–51*:*01*	-	04.66	-	03.40	01.62^f^	-	-	-
*23*:*01–50*:*01*	-	04.90	-	-	-	-	02.97	-
*24*:*02–08*:*01*	-	04.75	-	-	-	-	-	-
*29*:*01–45*:*01*	01.79	-	-	-	-	-	-	02.10
*29*:*02–44*:*03*	-	-	-	02.70	-	-	-	-
*30–18*	-	-	01.50	-	02.60	03.00	-	-
*30*:*02–53*:*01*	-	03.48	-	-	-	-	-	-
*32*:*01–40*:*02*	00.80	-	-	-	-	05.66	-	-
*33*:*01–14*:*01*	-	-	02.50	-	01.86^e^	01.41	-	-
*34*:*02–08*:*01*	02.12	-	-	-	-	06.11	-	02.10

^a^*02*:*01–44*:*02*.

^b^*02*:*01–44*.

^c^*02-44*.

^d^*02-50*.

^*e*^*33-14*.

^*f*^*02-51*.

#### *HLA-DRB1-DQB1* haplotypes

The most frequent *DRB1-DQB1* haplotypes with significant LD in Arabs are listed in Table 7. In general, class II haplotype frequencies are markedly higher than those of class I haplotypes. DRB1*03:01-DQB1*02:*01* haplotype was the most frequent *DRB1-DQB1* haplotype in Arabs (Table 7), and its frequency ranging from 3.2% in Lebanese to 16.60% in Tunisians. DRB1*03:01-DQB1*02:*01* is a common class II haplotype in the Mediterranean basin, and is frequent among Basques (17.5%) [41], Moroccans (17.3%) [25], Algerians (11.3%) [67], and Cretans (7.4%) [69]. In addition, DRB1*07:01-DQB1*02:*02* is also frequent in Arabs, such as Moroccans (16.70%), and is reportedly common in Spaniards (17.3%) [41], and Moroccans (12.6%) [25], but rare in Southern Tunisians (2.10%) (Gabesians). In addition, DRB1*07:01-DQB1*02:*01* is also a common *DRB1-DQB1* haplotype, and its frequency exceeds 4% in several Arab populations.

**Table 7:** Most frequent (%) *HLA* Class II (*DRB1-DQB1*) two-locus haplotypes with significant linkage disequilibrium (P<0.05) in Arabs.

*HLA-DRB1-DQB1*	Tun	Sau-B	Mor-Ch	Bah	Leb	Alg	Lib-J	Yem-J	Ber-Z	Ber-J
*01*:*02–05*:*01*	02.40	02.85	-	-	-	08.00	02.10	0.70	09.85	04.50
*07*:*01–02*:*02*	14.80	12.32	16.70	-	-	-	24.70^a^	22.10^a^	16.03	-
*03*:*01–02*:*01*	16.60	13.56	12.30	12.02	03.21	11.30	05.60^a^	12.00^a^	11.26	-
*10*:*01–05*:*01*	03.80	03.80	-	01.35	04.90	00.30	00.80	04.00	01.41	03.30
*07*:*01–02*:*01*	-	-	-	09.38	04.20	09.90	-	-	-	11.00
*15*:*01–06*:*02*	07.80	03.80	08.90	-	-	09.90	-	-	11.26	02.00
*04*:*02–03*:*02*	02.60	-	06.20	-	-	04.20	03.00	07.50	05.15	-
*13*:*01–06*:*03*	02.40	-	-	-	-	03.30	07.70	05.40	05.63	01.80
*16*:*01–05*:*01*	-	-	-	13.18	03.79	-	-	-	-	-
*04*:*01–03*:*02*	-	-	-	02.78	14.16	-	-	-	-	-
*11*:*01–03*:*01*	07.20^b^	02.22	-	11.98	31.42	04.70	09.30	03.40	07.00^b^	03.20

^a^DQB1*02

^b^*11*:*01/04-03*:*01*

In addition, DRB1*16:01-DQB1*05:*01* and DRB1*04:01-DQB1*03:*02*, rare in neighboring populations and Mediterraneans, were identified only in Lebanese and Bahraini Arabs. The high frequency of DRB1*11:01-DQB1*03:*01* haplotype (31.42%) among Lebanese is noteworthy, since it is the highest in all populations studied, but rare in Saudi (2.2%). Furthermore, DRB1*11:01/04-DQB1*03:*01*, identified in Arabs, is also frequent in Cretans (18.5%) [69] and Basques (3.1%)[41], while DRB1*01:02-DQB1*05:*01* was seen in Spaniards (6.30%) [41]. Varied frequency of DRB1*13:01-DQB1*06:*03* was also reported for Spaniards (13.23%) [86], Cretans (3.3%) [69], and Germans (10.8%) [87]. Likewise, DRB1*15:01-DQB1*06:*02* was observed in Cretans (2.6%) [65], German population (25.2%) [87], and Southern Ireland (14.90%) [23].

#### *HLA* class I and class II extended haplotypes

Table 8 shows the most frequent extended haplotypes in Arab populations, and their likely origins. The systematic review did not reveal haplotypes shared by Arab populations because of partial presentation of haplotypic data, disparity in the level of typing resolution, variability of the studied loci, and lack of data. In addition, Arab populations share their frequent extended haplotypes with several European, especially Mediterranean, and Asian populations (Table 8). Furthermore, the possible origins of the most frequent extended haplotypes among Arabs are mainly European, Asian or Autochthonous.

**Table 8:** The most frequent (%) *HLA* extended haplotypes in Arabs.

*HLA* Extended haplotypes	Arab Populations [references]	Possible origin
A*02:01-B*50:01-DRB1*07:01-DQB1*02:*02*^*a*^	Southern Tunisians (3.2%)[62], Berbers of Zrawa (8.12%) [24]	Euro-Asiatic
A*02:01–B*44– DRB1*04:02–DQB1*03:*02*^*b*^	Berbers of Zrawa (6.5%)[24] Tunisians (0.6%) [61]	Western European
A*24:02-B*08:01-C*07:02-DRB1*03:*01*^*c*^	Saudis (3.16%) [49]	Euro-Asiatic
A*23:01-B*50:01-C*06:02-DRB1*07:*01*	Saudis (3.16%) [49]	Autochthonous
A*33-C*8-B*14-DRB1*01:02-DQA1*01:01-DQB1*05:*01*^*d*^	Algerians (1.5%) [88]	Mediterranean
A*30-C*5-B*18-DRB1*03:01-DQA1*05:01-DQB1*02:*01*^*e*^	Algerians (1.5%) [88]	Iberian-paleo-North African
A*02:01-C*06:02-B*50:01-DRB1*07:01-DQA1*02:01-DQB1*02:*02*^*f*^	Moroccans (2.9%) [65]	Euro-Asiatic
A*01:01-C*06:02-B*50:01-DRB1*03:01-DQA1*05:01-DQB1*02:*01*^*g*^	Moroccans (2.9%) [65]	Mediterranean
A*30-B*07-DRB1*03-DQA1*05:01-DQB1*02:*01*^*h*^	Jordanians (1.38%) [31]	Euro-Asiatic
A*1-B*8-DRB1*03-DQA1*05:01-DQB1*02:*01*^*i*^	Jordanians (1.03%) [31]	Pan-European
A*02:01-B*50:01-DRB1*07:*01*^*j*^	Libyans (4.24%) [32] Tunisians (1.8%) [60], and Ghannouch (2.5%) [33].	North African
A*11:01-B*52:01-DRB1*15:*02*^*k*^	Libyans (2.54%) [32]; Yemen Jews (0.93%) [23]	Mediterranean
A*69-B*49-DRB1*04:03-DQB1*03:*02*	Palestinians (2.4%) [29]	Autochthonous
A*24-B*18-DRB1*11:04-DQB1*03:*01*^*l*^	Palestinians (1.8%) [29]	Central-South-Eurasian

^a^ present in Spaniards (1.2%) [41], Turks (1.3%) [79], Italians (0.5%) [68], and Moroccan Jews (2%) [66].

^b^ also found in British (2.6%), Cornish (7.9%), Danes (2%) [39], Italians (0.9%) [68], Spaniards (0.6%) [41], Spanish Basques (1.9%), Pasiegos (3.3%), Cabuemigos (2.2%) [77], and Portuguese (3.1%) [39].

^c^ present at low frequencies in the Euro-Asian minorities of Germany [23].

^d^ found in Armenians (0.031), Sardinians (0.027), French (0.014), Greeks (0.011), and Italians (0.007) [68].

^e^ also found in Sardinians (11.4%), and French-Basques (4.7%) [68].

^f^ present also in Mongolians [68], Turks [79].

^g^ found in Spaniards, Italians, and north Africans [65].

^h^ present in Cornish (0.084), British (3.3%), and Danes (3.8%) [68].

^i^ present in Basques (5%), Spaniards (3.4%) [41], Macedonians (4.9%) [78], Yugoslavians (7.7%), British (2.9%), and Germans (4.8%) [68].

^j^ found in Poland Jews (1.15%); Ashkenazi Jews (0.92%) [23].

^k^ present in Ashkenazi Jews (1.05%) [23].

^l^ found in Armenians (2.1%) and Italians (0.7%) [23].

## Discussion

This meta-analysis is the first genetic anthropology study in MENA region, and included 100 populations from 36 Arab and neighbouring countries, and comprising in excess of 16,000 individuals. A main outcome of the study is the lack of striking differences in the distribution of *HLA* alleles and haplotypes between North Africans and Arabian Peninsula populations. On the contrary, key differences were noted between Levant Arabs (Lebanese, Palestinians, Syrians), and other Arab populations, highlighted by high frequencies of A*24, B*35, DRB1*11:*01*, DQB1*03:*01*, and DRB1*11:01-DQB1*03:*01* haplotype in Levantine Arabs compared to other Arab populations. Class I haplotype frequencies are lower than Class II haplotypes, because of weak LD between *A* and *B* loci, due to long physical distance between them, compared to *DRB1* and *DQB1* loci. The identification of shared haplotypes between Arabs and other Mediterranean and Asian populations is attributed to the higher admixture of Mediterraneans and Asians in Arab populations.

### Iberians, North Africans, and Arabian Peninsula inhabitants

The relatedness between North Africans and Iberians was previously discussed [29, 59–62, 69, 78, 79, 86, 88]. Using correspondence analysis, NJ trees and genetic distances, our results show that North Africans are genetically close to Iberians, which is supported by historical events. First, this relatedness is attributed to the Berber migration from the African Sahara northwards in 10000–4000 BC, because of hyper-arid conditions [69]. It may also be explained by the similar history between Iberians and North Africans, both of whom were invaded by Phoenicians, Romans, Germans, Muslim Arabs [89]; the respective invading armies had a mixed genetic complexity; indeed, most of them were mercenaries recruited in recent conquests like in the case of Phoenicians [90] and Muslim who invaded Iberia had troops that were mostly Berbers. The invasion of Iberia by Muslims in the 8th century AD may have had a role in the relatedness between North Africans and Iberians for two reasons: first, most Muslim invaders recruits were North African Berbers, and the second is explained by the 8 centuries period of settlement of the Muslims in Iberia, although more ancient and continuous gene exchange since prehistoric times between Iberia and North Africa may have been induced the main exchange [86]; massive mixed marriages and breeding across religious Iberian groups under Muslim rule is not documented.

The analyses performed showed that current North Africans are closely related to Tunisian (Zrawa and Matmata) and Moroccan (Sousse-Agadir and Eljadida) Berbers, suggesting that North Africans have a genetic Berber profile. On the contrary, North Africans displayed a greater distance from the Arabs of Levant (Palestinians, Syrians, Lebanese, and Jordanians), indicating low genetic contribution of Phoenician and Levant Arab invasion of North Africa. These observations based on *HLA* markers prompted the conclusion that all Berbers of North Africa constitute a homogeneous genetic unit, except for small isolates, such as the Berbers of Djerba, who display a Berber genetic profile.

Saudi populations used in this study originated from Eastern Saudi Arabia, especially from Riyadh province. There is no reliable *HLA* data on Eastern Saudi Arabia that shed light on pre-Islamic history; some ancient people may have originated from old Persians, but quantification is difficult and undetermined [91]. The genetic heterogeneity between Eastern and Western Saudi Arabia is very possible, and should be taken into account in further interpretation. All analyses performed here, using *HLA-A*,*-B*, *-DRB1*, and *DQB1* markers support the notion that Saudis along with the Kuwaitis and Yemenis are closely related to North Africans.

The most plausible explanation for West Arabia and Yemen clustering with Iberian/North Africans is a possible important massive migration that occurred when Sahara underwent desiccation in all directions [92, 93]. Cultural and language relatedness of many Mediterranean languages, including old Iberian and Basque [92], with Berber language are concordant with our genetic findings and Saharan origin hypothesis; also a part of Arabian Peninsula inhabitants (including Yemen) may had been reached by Saharan people. In fact, Malika Hachid who has been studying Saharan and North African Archaeology, culture and rock painting/writing of prehistoric Sahara, even suggests that first known writing alphabet was originated in Sahara. Proto-Berber writing rock characters have been used (very similar to present day used Berber scripts). This Proto-Berber language could have appeared 5,000 years BC [94, 95].

Explanation to *HLA* Kuwait genetic similarity to this group seems more difficult to achieve but interaction between Arabian Peninsula and Mesopotamia through this strategic Kuwait area is documented since 6,500 years BC (Ubard Period) [96].

### Arabs of Levant

Using genetic distances, correspondence analysis and NJ trees, we showed earlier [61, 62] and in this study that Palestinians, Syrians, Lebanese and Jordanians are closely related to each other and to Eastern Mediterranean Europeans (Turks, Cretans, Greeks), Egyptians and Iranians, and confirmed by *HLA* class I (*A*, *B*) and class II markers (*DRB1* and *DQB1*) analysis. However, Levant Arabs are distant from North African Arabs (Tunisians, Algerians, Moroccans and Libyans) and Iberians (Basques, Spaniards). The strong relatedness between Levant Arab populations is explained by their common ancestry, the ancient Canaanites, who came either from Africa or Arabian Peninsula via Egypt in 3300 BC [97], and settled in Levant lowlands after collapse of Ghassulian civilization in 3800–3350 BC [98]. The relatedness is also attributed to the close geographical proximity, which constituted one territory before 19th century British and French colonization.

The close relatedness of Levant Arabs to Egyptians, as confirmed genetic distances using *HLA* markers, may be due to three reasons. First, Egypt is a neighbor to Levant Arab countries, and historically part of the Levant. Second, the Egyptians invaded the Levant several times throughout history; the most significant was 1468 BC invasion, where they settled for 12 centuries [99]. Third, the Canaanites, the likely ancestors of Levant Arabs, may have originated from Africa through Egypt, where they settled for a long period, suggesting likely admixture between Canaanites and Egyptians.

Historically, Levant is a wider region that included countries along the Eastern Mediterranean with its islands, and extended from Greece to Cyrenaica [100]. Broadly, Levant was historically characterized by high migratory flow between its sub-regions in all directions. For example, present-day Levant comprising Palestine, Lebanon, Syria, and Jordan has undergone successive invasions by populations originating from the great Levant, including Egyptians (1468 BC), Horites, Amorites, Hitites (Turks), Greeks (1200 BC), Assyrians (1090 BC) [99], and more recently the Ottomans. This has favored admixture, reduced distances and homogenized Great Levant populations, thus explaining the close relatedness of Levant Arabs to Eastern Mediterranean populations. On the other hand, Levant Arabs are distant from Saudis, Kuwaitis, and Yeminis, an indication that the contribution of the Arabian Peninsula populations to Levantine gene pool is low, probably due to the absence of the demographic aspect of 7th century invasion.

### Sudanese and Comorians

Sudanese are close to sub-Saharan Africans (Nigerians, Congolese, and Senegalese), and North Africans, in particular Egyptians, suggesting that the genetic profile of Sudanese is the admixture between North Africans (especially Egyptians) and sub-Saharan Africans throughout history. The close relatedness of Sudanese to sub-Saharan Africans suggests a reduced genetic effect of Arabs on Sudanese. Also, the Comorians (Comoros islands officially joined League of Arab Countries in 1993) are close to sub-Saharan Africans (Congolese, Nigerians, and Gabonese) [43], Egyptians, Iranians, and Eastern Mediterranean. This suggests high admixture between populations belonging to three continents in the Comoro Islands, and can be explained by their geographical position as a corridor for international trade.

### Bahrainis, Emiratis, and Omanis

Bahrainis, Emiratis, and Omanis are geographically similar populations, which explains their genetic relationship as demonstrated in this study. These three populations tend to form a heterogeneous group with Pakistanis, Indians, Iranian Arabs (Famoori), Sardinians (the later probably close to Iberians/North Africans but behaving as out layer group in analyses because of they are a genetic island isolate), Egyptians, and some sub-Saharan Africans, such as Congolese. These populations appear close to certain Eastern Mediterranean populations including Greeks, Macedonians, and those further, in particular North Africans, hence explaining their intermediate grouping, and distinction from two main clusters. Collectively, this suggests high admixture in these populations brought about by their commercially important position. Sardinia is a relative genetic isolate “founded” by Iberian Norax/Nora (first documented Sardinian capital close to Cagliari) and Iberians/North Africans may be genetically related to Sardinians (A*30-B*18-Cw*5 basic *HLA* haplotype is very high in Sardinia, Iberia, and North Africa) [93].

### Minorities of Arab World

#### Ethnic minorities

The Kurds and Berbers are the two major ethnic minorities in Arab world. Berbers are indigenous North African ethnic group found over a vast area stretching from Atlantic Ocean to Siwa Oasis in Egypt, and from Mediterranean Sea to Niger River. Berbers number about 20 million people, and constitute 40–45% of Moroccans, 20–25% of Algerians, and 2–7% in both Libya and Tunisia. The Kurds live in the northern regions of Iraq (15–20%) and Syria (10%). They constitute an Indo-European ethnic group, and speak Kurdish. Less important minorities include Armenians, Nubians, Assyrians, and Turkmen [99].

Berbers populations used in this work are closely linked to each other, as well as to present-day North Africans, and to Western Mediterranean populations, especially Iberians. Indeed, the Moroccan Berbers are not genetically different from the current Moroccans, nor those of neighboring populations, like Algerians and Tunisians. This also applies to Tunisian Berbers, except those of the island of Djerba, who appear to be related to Eastern Mediterranean populations, including Levant Arabs. This suggests that North African Berbers are in perfect harmony with their environments, and that differences between them are cultural rather than genetic due to 7th century Arabization of the region.

Clustering and genetic distances analyses demonstrated that Iraqi and Iranian Kurds are not genetically different from Iranians or neighboring populations, including Levant Arab, and are close to Turks and other Eastern Mediterranean populations. This suggests that Kurds originate from the region, and are in genetic harmony with neighboring populations, despite the clear cultural differences. This suggests that Kurds, Syrians, Jordanians, Palestinians, Iraqis, Lebanese, and Iranians probably share the same genetic profile, with few differences. Accordingly, our findings confirm the results of an earlier study of Arnaiz-Villena on Iraqi Kurds [54].

#### Religious minorities

Sunni Muslims constitute the majority (80%) of Arab populations, followed by Shi'a Muslims (10%) who are present in parts of Iraq, Lebanon, Saudi Arabia, Kuwait, Yemen, and Bahrain. Non-Muslims make up about 10% of all Arabs, and Christianity (6%) is the second largest religion among Arabs, with about 20 million Christians living in Lebanon, Egypt, Iraq, Syria, and Jordan. Other minor religions (4%) such as Judaism, Druze and others are practiced on a much smaller scale [99].

*HLA* data on Sunni and Shiite Arabs are not available, same as comparison of Muslims to Christians. The only available data are those concerning Arab Jews. In this study, data are available for three Jewish populations, including two from North Africa (Moroccan and Libyan Jews) and one from the Arabian Peninsula (Yemenite Jews). While genetic distances separating these three groups of Jews are small (S1 Table), genetic heterogeneity between these Jewish populations was noted. For example, Yemenite Jews are related to Western Mediterranean populations, including North Africans and Iberians, while Libyan Jews are related to Eastern Mediterraneans, including Levantine Arabs. The relatedness of Moroccan Jews depends to other communities on the studied *HLA* loci; they associate with Eastern Mediterraneans using *DRB1*, but group with Eastern Mediterraneans when the other markers are used.

## Conclusion

This study supports the notion that Arabs are divided into four groups. The first consisting of North Africans (Algerians, Tunisians, Moroccans, and Libyans), Saudis, Kuwaitis, and Yemenis, with relatedness to Western Mediterraneans, including Iberians. The second includes Levantine Arabs (Palestinians, Jordanians, Lebanese, and Syrians), Iraqi, and Egyptians, who appear to be related to the Eastern Mediterranean and Iranians, who in turn belonged to 'Great Levant' historically described. The third consists of Sudanese and Comorians who associate with Sub-Saharan Africans. Finally, the fourth group of Arabs comprises Omanis, Emiratis, and Bahrainis. This group associates with heterogeneous populations (Mediterranean, Asian and sub-Saharan). Lastly, the two main indigenous minorities, Berbers and Kurds, are not genetically different from the ‘host’ and neighboring populations.

## Supporting information

S1 Checklist PRISMA 2009 checklist. (DOC)

S1 Fig Neighbor-Joining dendrograms, based on standard genetic distances (SGD), showing relatedness between Arabs and other populations using generic HLA-DRB1* allele frequencies data. Populations’ data were taken from references detailed in Tables 1 and 2. Bootstrap values from 1.000 replicates are shown.
(TIF)

S2 Fig Neighbor-Joining dendrograms, based on standard genetic distances (SGD), showing relatedness between Arabs and other populations using generic HLA-B* allele frequencies data. Populations’ data were taken from references detailed in Tables 1 and 2. Bootstrap values from 1.000 replicates are shown.
(TIF)

S1 Table Genetic distances between three groups of Arab Jews based on HLA-DRB1 and -DQB1 alleles frequencies. (DOC)
