# Supplementary figures and images for: The genetic heterogeneity of Arab populations as inferred from HLA genes
Source: PLoS One. 2018 Mar 9;13(3):e0192269. doi: 10.1371/journal.pone.0192269 (PMC5844529; doi:10.1371/journal.pone.0192269)

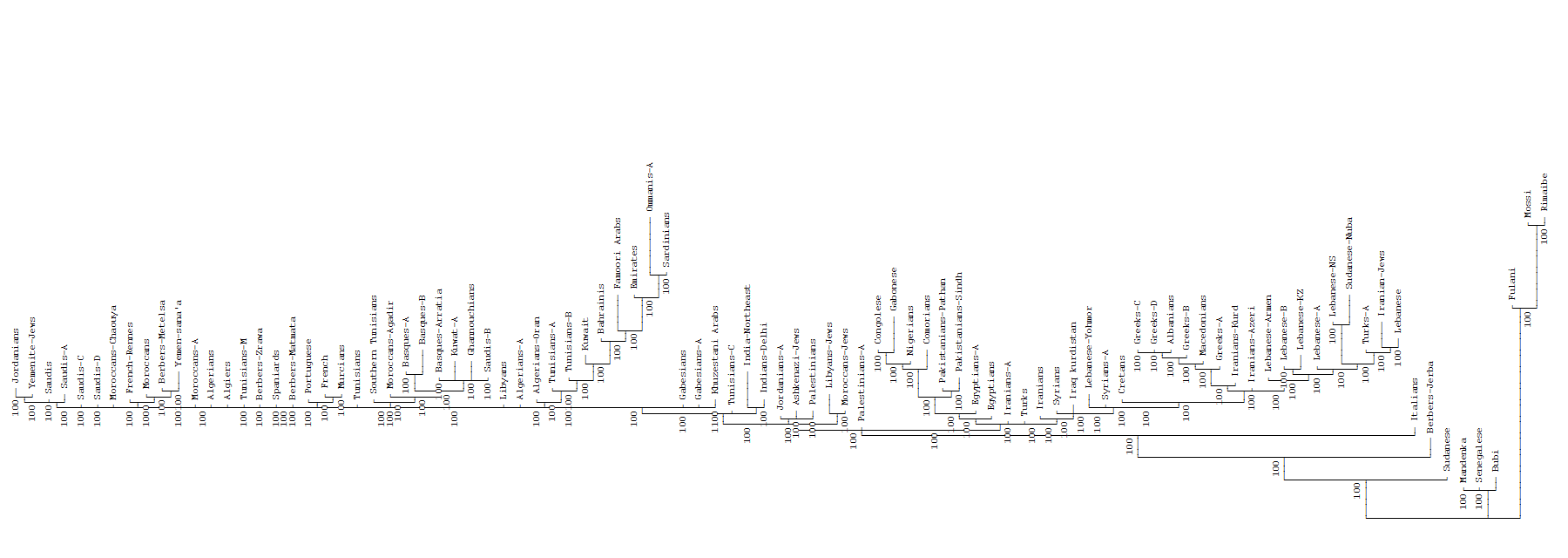

Supplement: S1 Fig — Populations’ data were taken from references detailed in Tables 1 and 2. Bootstrap values from 1.000 replicates are shown. (TIF) [file pone.0192269.s002.tif]

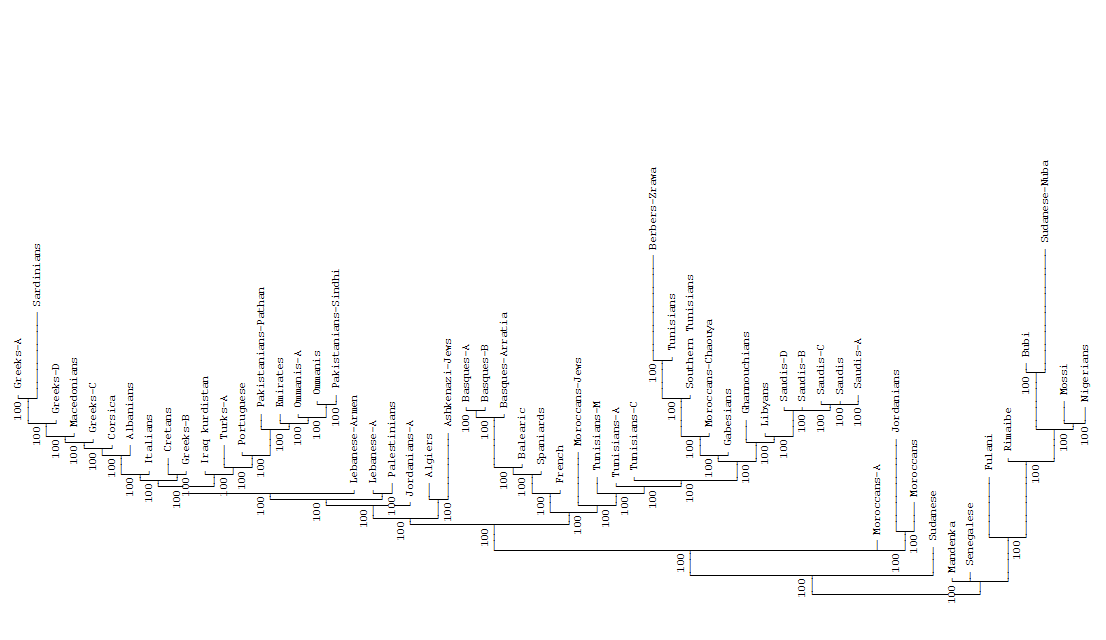

Supplement: S2 Fig — Populations’ data were taken from references detailed in Tables 1 and 2. Bootstrap values from 1.000 replicates are shown. (TIF) [file pone.0192269.s003.tif]
