# Supplementary material for: The genetic heterogeneity of Arab populations as inferred from HLA genes
Source: PLoS One. 2018 Mar 9;13(3):e0192269. doi: 10.1371/journal.pone.0192269 (PMC5844529; doi:10.1371/journal.pone.0192269)
Supplement: S1 Table — (DOC) [file pone.0192269.s004.doc]

|  | Moroccans-Jews | Yemenite-Jews | Libyans-Jews |
| --- | --- | --- | --- |
| Moroccans-Jews | 0.0000 | 0.0808 | 0.0726 |
| Yemenite-Jews | 0.0808 | 0.0000 | 0.0227 |
| Libyans-Jews | 0.0726 | 0.0227 | 0.0000 |

Genetic distances between three groups of Arab Jews based on HLA-DRB1 and -DQB1 alleles frequencies
